# Supplementary material for: Data leakage inflates prediction performance in connectome-based machine learning models
Source: Nat Commun. 2024 Feb 28;15:1829. doi: 10.1038/s41467-024-46150-w (PMC10901797; doi:10.1038/s41467-024-46150-w)
Supplement: Supplementary file 1 — Supplementary Information [file 41467_2024_46150_MOESM1_ESM.pdf]

Supplementary Materials for:

Data leakage inflates prediction performance in connectome-based machine learning models

S1. Summary of the leakage types in this study

**Leakage type**	**Why is it leakage?**	**Relation to Kapoor and Narayanan** [17]
Leaky feature selection	If the test data contributes to the feature selection process, there is no assessment of generalizability of the feature selection from training to test data.	Feature selection jointly on the training and test sets
Leaky site correction	If sites are controlled for in the training/test data simultaneously, then the test data indirectly leaks information back into the model training via the site correction parameters.	Pre-processing on the training and test sets
Leaky covariate regression	If covariates are regressed in the training/test data simultaneously, then the test data indirectly leaks information back into the model training via the covariate regression parameters.	Pre-processing on the training and test sets
Family leakage	Having one family member in the training dataset and another in the test dataset breaches the independence between training/test data. This could be particularly relevant for heritable behaviors.	Non-independence between the training and test sets;<br>Sampling bias
Subject leakage	Similar to family leakage, if subjects are repeated across the training and test sample, then the model training is directly affected by a subject that can also be in the test dataset.	Duplicate data points

Supplementary Table 1. Summary of the leakage types used in this study and their mapping to those used by Kapoor and Narayanan [17].

S2. Evaluation of leakage with q^2 in the main dataset (HCPD)

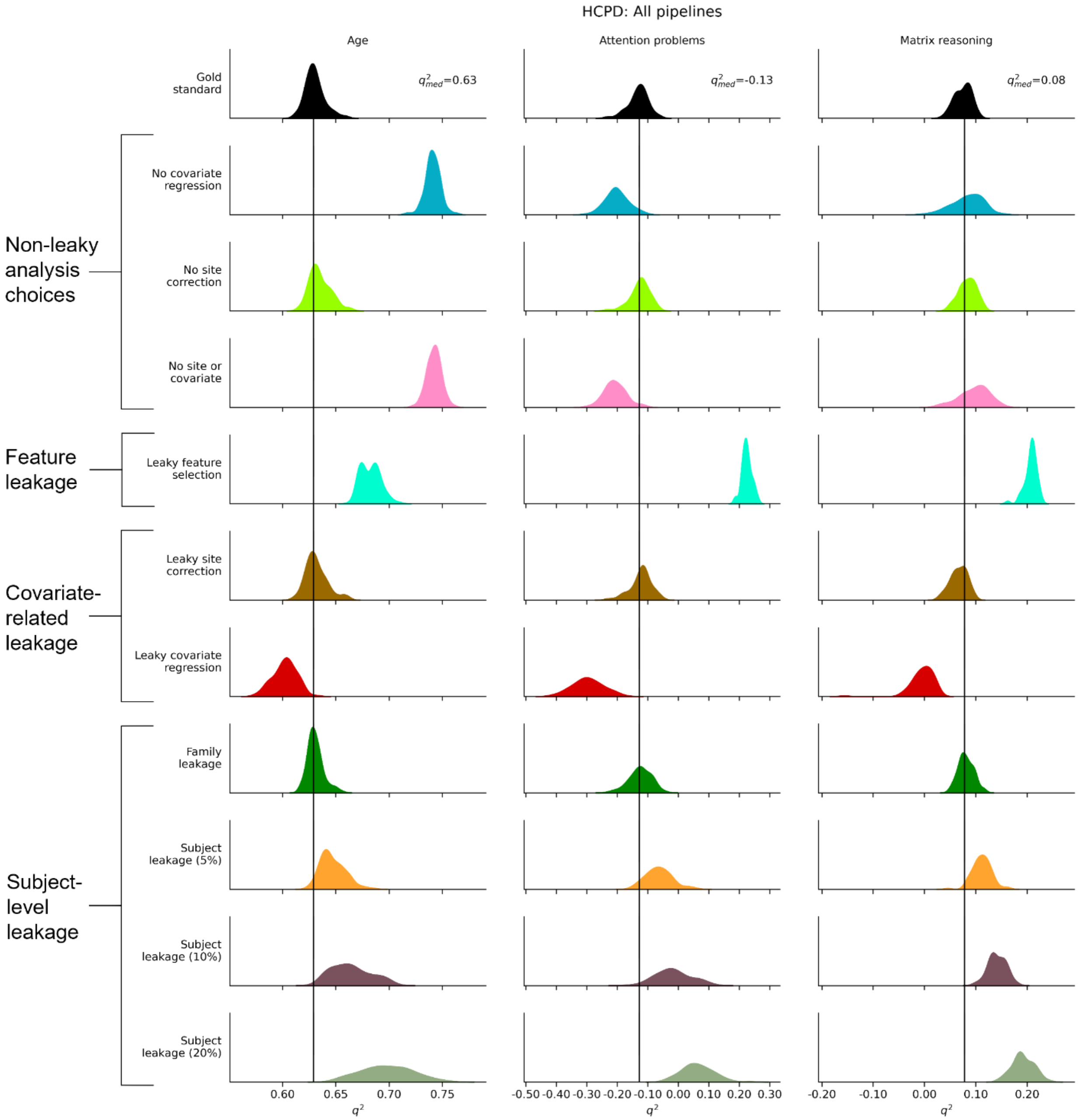

Supplementary Figure 1. Evaluation of leakage in HCPD with a metric of q^2, related to Figures 2-5. Rows represent different leakage types, and columns show different phenotypes. The black bar represents the median performance of the “gold standard” models across random iterations, and the histograms show prediction performance across 100 iterations of 5-fold cross-validation. HCPD: Human Connectome Project Development.

S3. Family leakage analysis

Beyond the three original phenotypes and models in this study, we considered several additional phenotypes from the Child Behavioral Checklist (CBCL) [56], as well as one additional model (Random Forest). The phenotypes were the Anxiety and Depression CBCL Syndrome Scale Raw Score (Anx/Dep), Aggressive CBCL Syndrome Scale Raw Score (Aggression), Internal CBCL Syndrome Scale Raw Score (Internal), and the External CBCL Syndrome Scale Raw Score (External). The Random Forest was considered in case the other models (ridge regression, SVR, CPM) had too low of a memorization capacity that would limit the effects of leakage. For the Random Forest [27], 10 estimators were used, and a grid search was performed varying the maximum depth (3, 5, 7, 9).

We found that, across all models and phenotypes, the median performance of twin leakage was greater than the median gold standard performance (Figure S2). The effects of leakage were greater for ridge regression and SVR compared to CPM and the Random Forest. Although not tested, family leakage may become increasingly impactful for complex models, such as neural networks, that are trained with larger datasets and more participants per family.

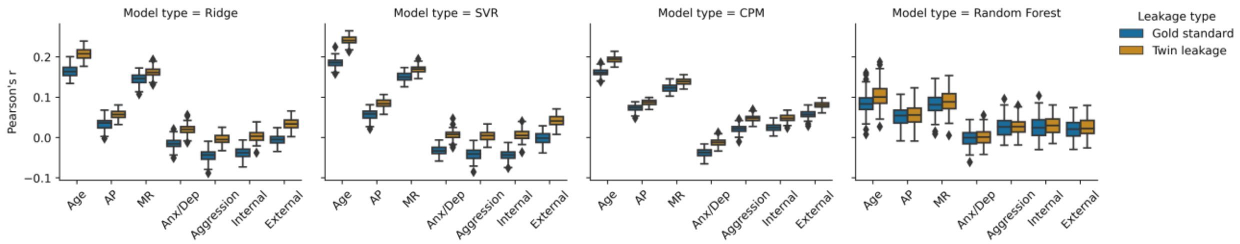

Supplementary Figure 2. Comparison of prediction performance between the gold standard and twin leakage in the ABCD twin subset, related to Figure 6. Boxplot elements were defined as follows: the center line is the median across 100 random iterations; box limits are the upper and lower quartiles; whiskers are 1.5x the interquartile range; points are outliers. Four models (ridge regression, SVR: support vector regression, CPM: connectome-based predictive modeling, Random Forest) and seven phenotypes were included. In all cases, the median performance was higher for twin leakage compared to the gold standard. AP: Attention CBCL Syndrome Scale Raw Score; MR: WISC-V Matrix Reasoning Total Raw Score; Anx/Dep: Anxiety and Depression CBCL Syndrome Scale Raw Score; Aggression: Aggressive CBCL Syndrome Scale Raw Score; Internal: Internal CBCL Syndrome Scale Raw Score; External: External CBCL Syndrome Scale Raw Score.

We compared the similarity of each phenotype between twins and the corresponding increase in prediction performance in a leaky vs. non-leaky pipeline. As a metric of similarity, we took the ratio of the mean absolute error of the phenotype between each twin pair to the mean absolute error between the participant and all non-twin participants, and this ratio was averaged across all participants. The MAE ratio for participant *p* is defined as:

$$MAE\ Ratio\ = \frac{MAE(y_p\ , y_{p,twin})}{MAE(y_p\ , y_{p,non-twin})}$$

Thus, a value closer to 0 reflects greater similarity of that phenotype between twins. We chose to use phenotype similarity instead of literature estimates of heritability because a phenotype such as age is not necessarily “heritable,” but it is identical (or nearly identical due to different interview dates) for twins. Similarly, the CBCL measures were determined by a parent questionnaire and thus may reflect the tendencies of a parent in answering questions rather than explicit heritability of a trait.

The most similar phenotypes did not necessarily show greater leakage effects (Figure S3). This result could point toward the limited memorization capacity of the model, given the study design. For example, if there were many members per family, the model may more easily "memorize" the signature of family members, and the effects of leakage may be greater.

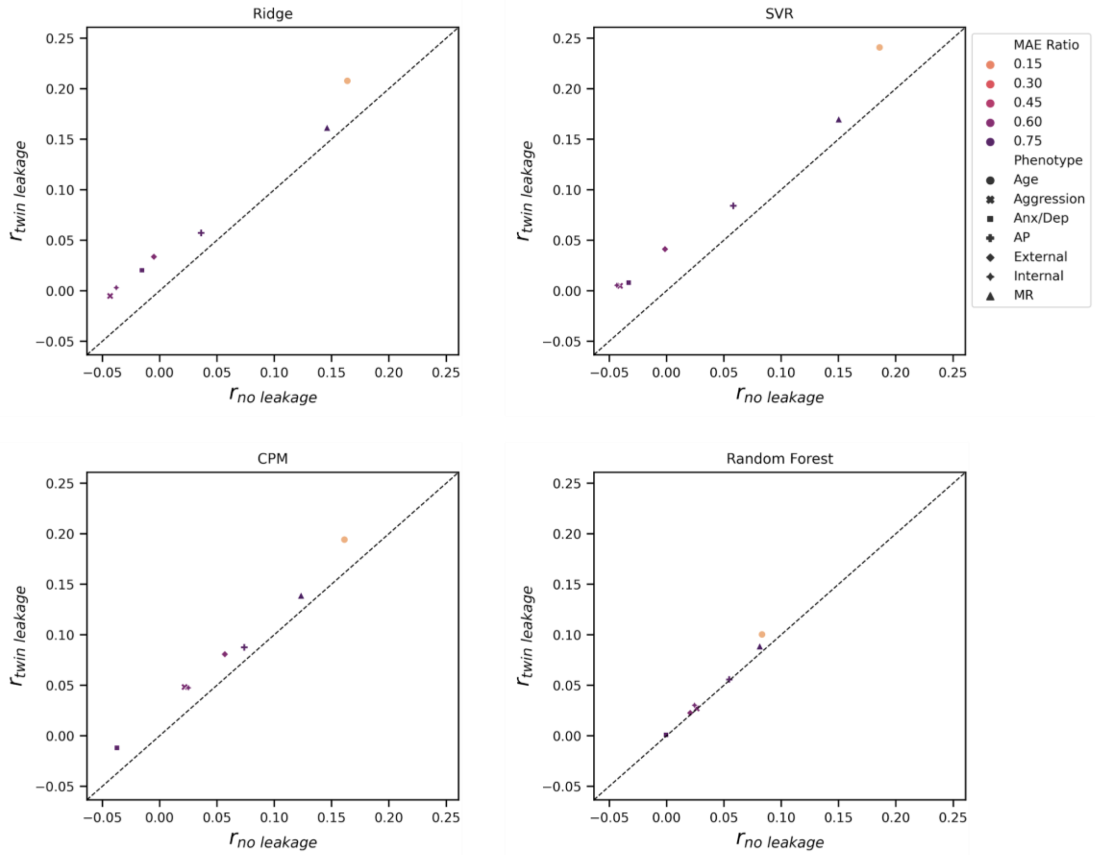

Supplementary Figure 3. Comparison of prediction performance with twin leakage (*y*-axis) and the gold standard (*x*-axis) colored by phenotype similarity, related to Figure 6. An MAE Ratio closer to zero entails greater similarity between twins. The shape of each point indicates the phenotype. AP: Attention CBCL Syndrome Scale Raw Score; MR: WISC-V Matrix Reasoning Total Raw Score; Anx/Dep: Anxiety and Depression CBCL Syndrome Scale Raw Score; Aggression: Aggressive CBCL Syndrome Scale Raw Score; Internal: Internal CBCL Syndrome Scale Raw Score; External: External CBCL Syndrome Scale Raw Score.

Furthermore, we performed a simulation that altered the percentage of one-individual families in the dataset. To do this, we started with only families with multiple individuals, and then we added in random fractions of the participants without family members. In general, the effects of leakage increased as the fraction of participants coming from families with multiple members increased (Figure S4). However, the effects were still relatively small.

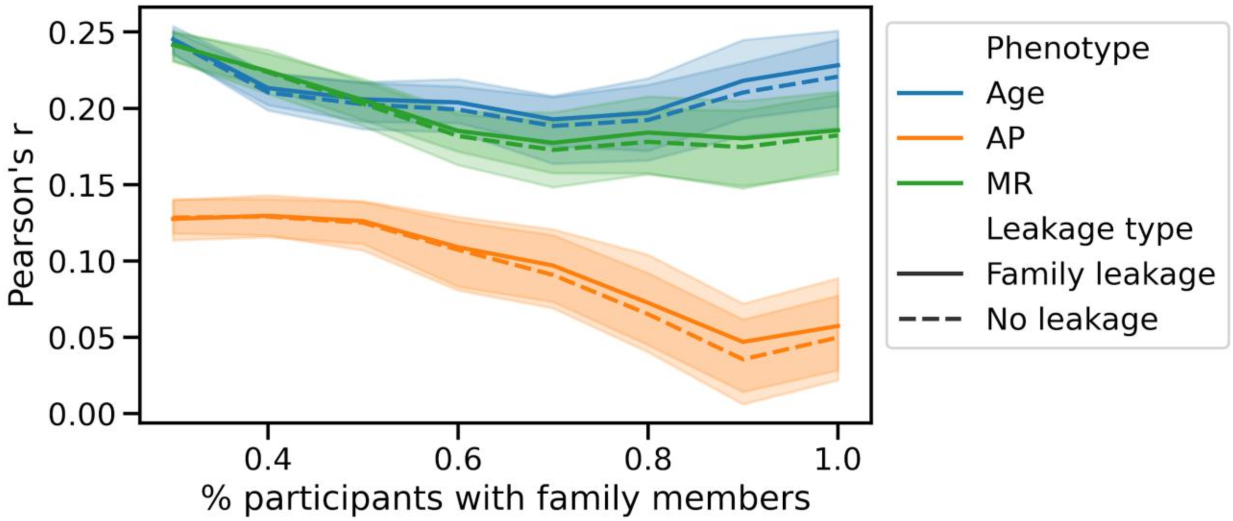

Supplementary Figure 4. Simulation varying the percentage of participants with family members in the ABCD datasets, related to Figure 6. Ridge regression was performed for 100 random iterations with the datasets subsample consisting of 30, 40, 50, 60, 70, 80, 90, and 100% multi-participant families (100% is restricted to only participants with other family members). The error bars reflect the 2.5th and 97.5th percentiles of the 100 random iterations. AP: Attention Problems, MR: Matrix Reasoning.

S4. Leakage for additional datasets and models

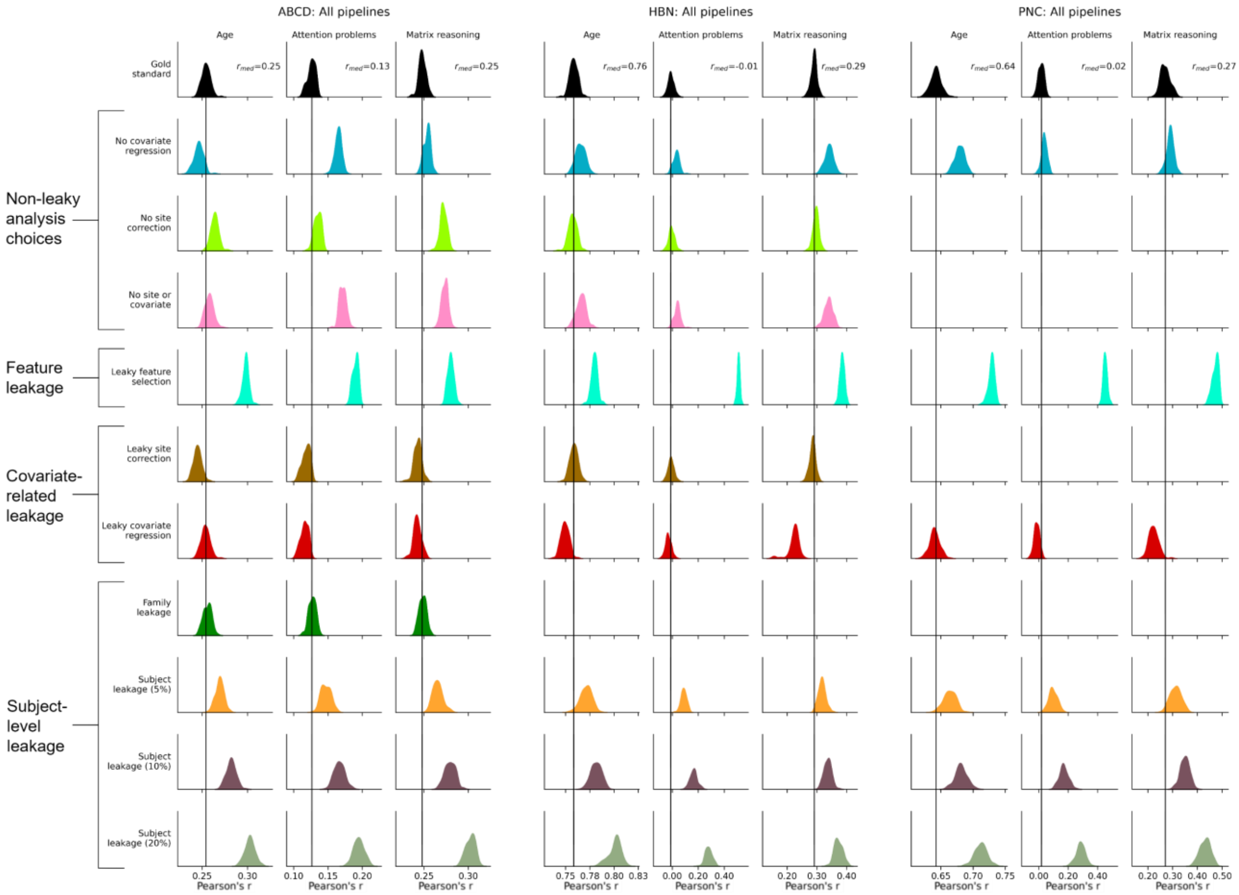

Supplementary Figure 5. Summary of leakage types in additional three datasets (ABCD, HBN, PNC), related to Figure 7. The rows show different leakage types, and, within each dataset, the columns show different phenotypes. The black bar represents the median performance of the “gold standard” models across random iterations, and the histograms show prediction performance across 100 iterations of 5-fold cross-validation. ABCD: Adolescent Brain Cognitive Development; HBN: Healthy Brain Network; PNC: Philadelphia Neurodevelopmental Cohort.

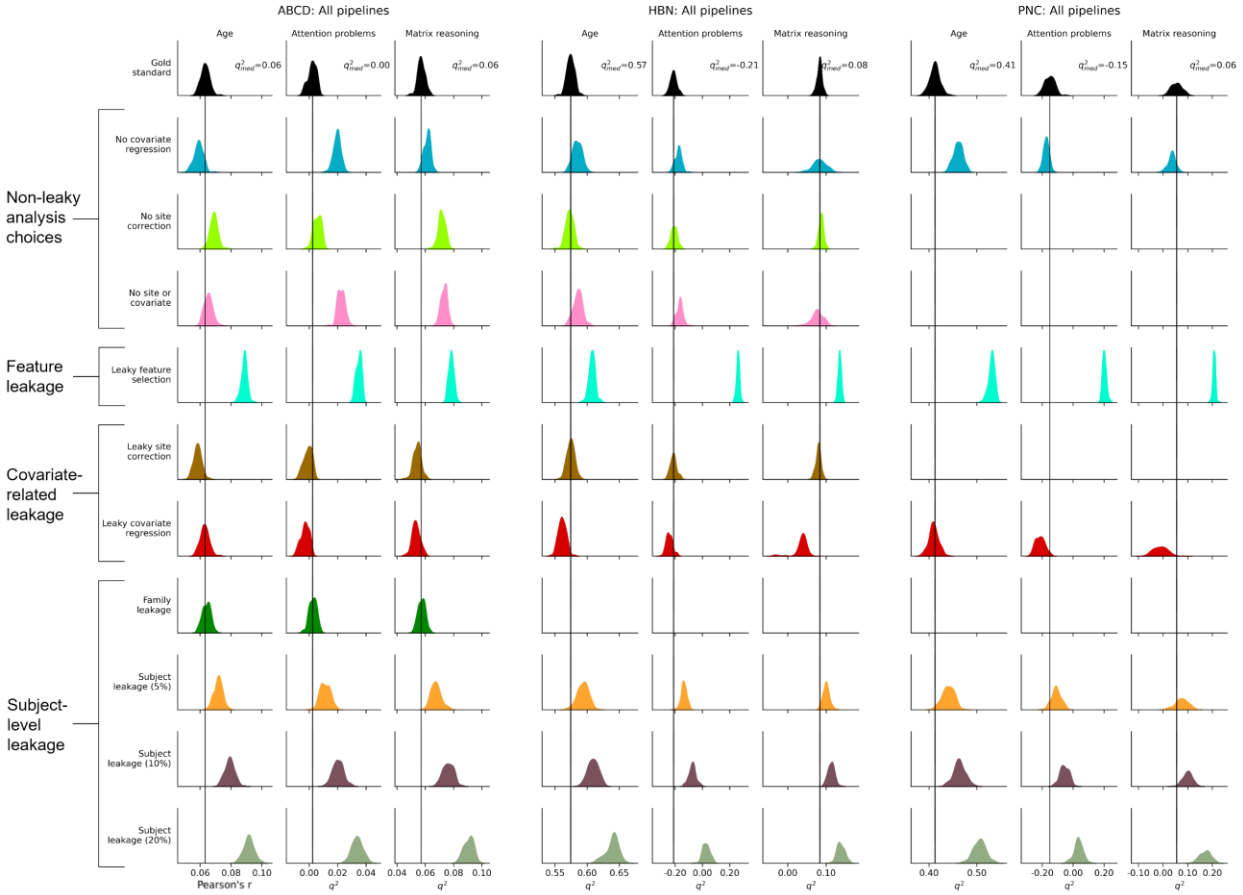

Supplementary Figure 6. Evaluation of leakage in ABCD, HBN, and PNC with a metric of q^2, related to Figure 7. Rows represent different leakage types, and columns show different phenotypes. The black bar represents the median performance of the "gold standard" models across random iterations, and the histograms show prediction performance across 100 iterations of 5-fold cross-validation. ABCD: Adolescent Brain Cognitive Development; HBN: Healthy Brain Network; PNC: Philadelphia Neurodevelopmental Cohort.

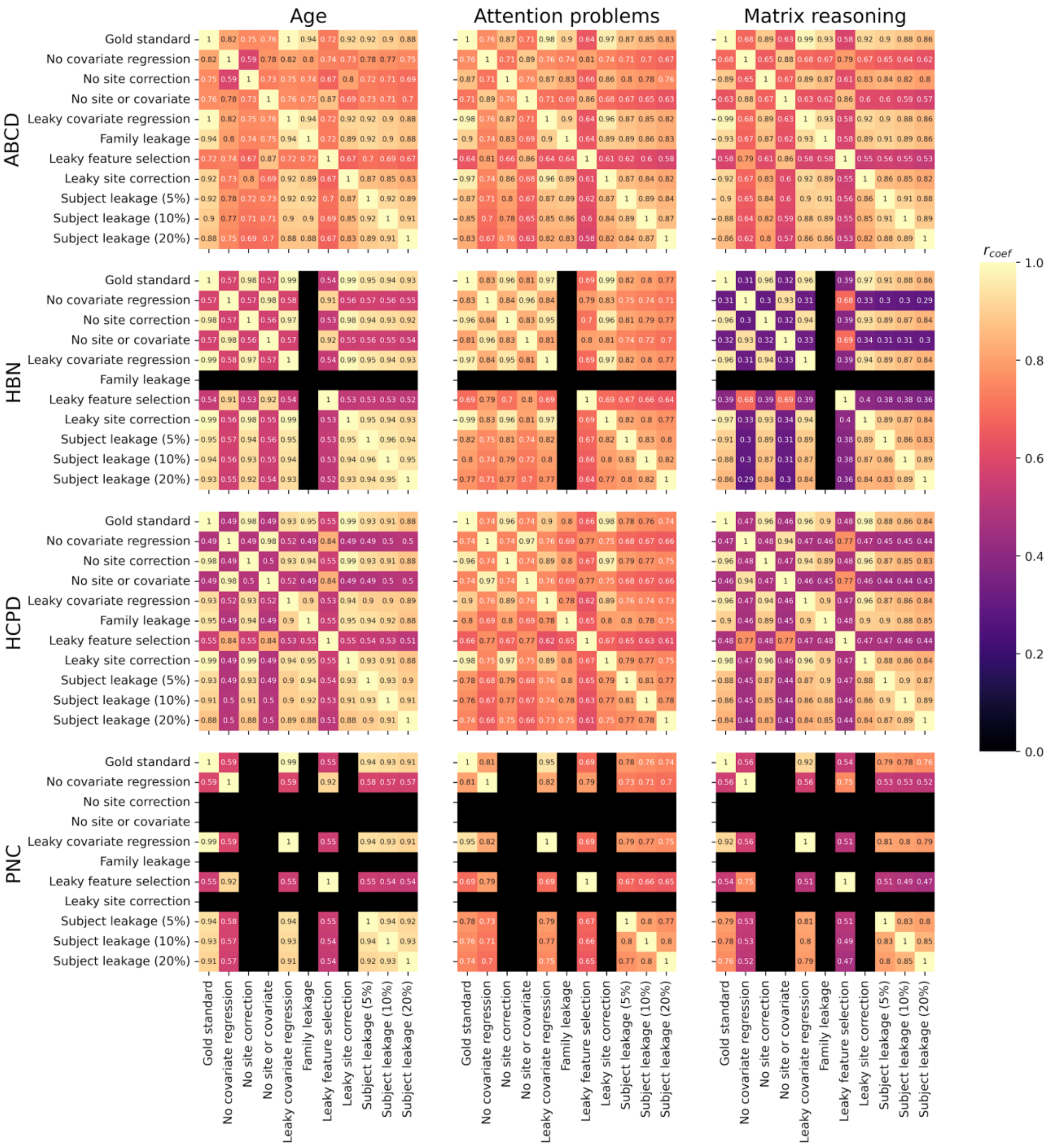

Supplementary Figure 7. Similarity of coefficients across all pipelines, averaged over 100 random seeds, related to Figure 8. For each pair of the 13 pipelines, we computed the correlation between their coefficients. Missing values (i.e., no site information in PNC) are shown in black. ABCD: Adolescent Brain Cognitive Development; HBN: Healthy Brain Network; HCPD: Human Connectome Project Development; PNC: Philadelphia Neurodevelopmental Cohort.

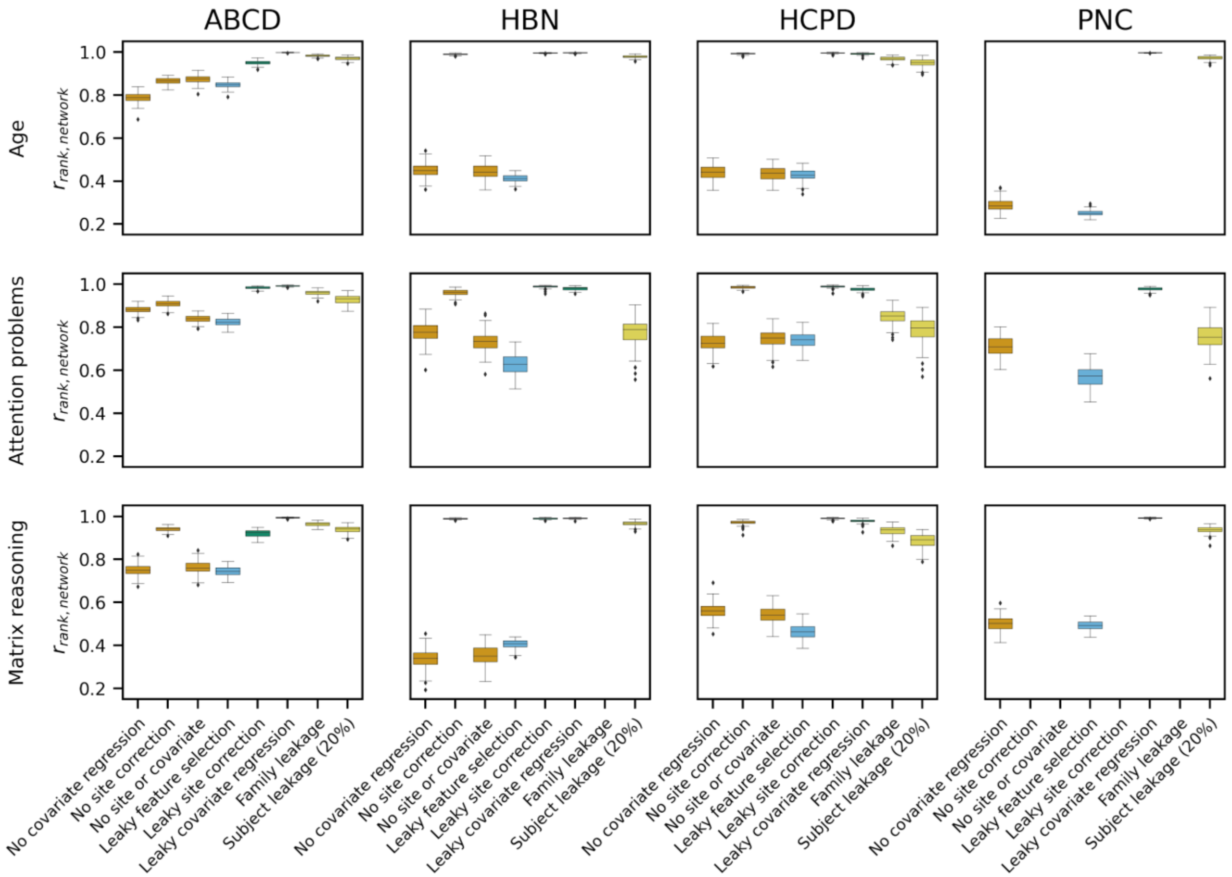

Supplementary Figure 8. Subnetwork-level rank correlations for number of selected features, related to Figure 8. The boxes are colored by the leakage family: black (gold standard), orange (non-leaky analysis choices), blue (feature leakage), green (covariate-related leakage), yellow (subject-level leakage). To compare the gold standard features to features from the other pipelines, we calculated the rank correlation between the number of features in each subnetwork (adjusted for subnetwork size). Boxplot elements were defined as follows: the center line is the median of the rank correlation across 100 random iterations; box limits are the upper and lower quartiles; whiskers are 1.5x the interquartile range; points are outliers. ABCD: Adolescent Brain Cognitive Development; HBN: Healthy Brain Network; HCPD: Human Connectome Project Development; PNC: Philadelphia Neurodevelopmental Cohort.

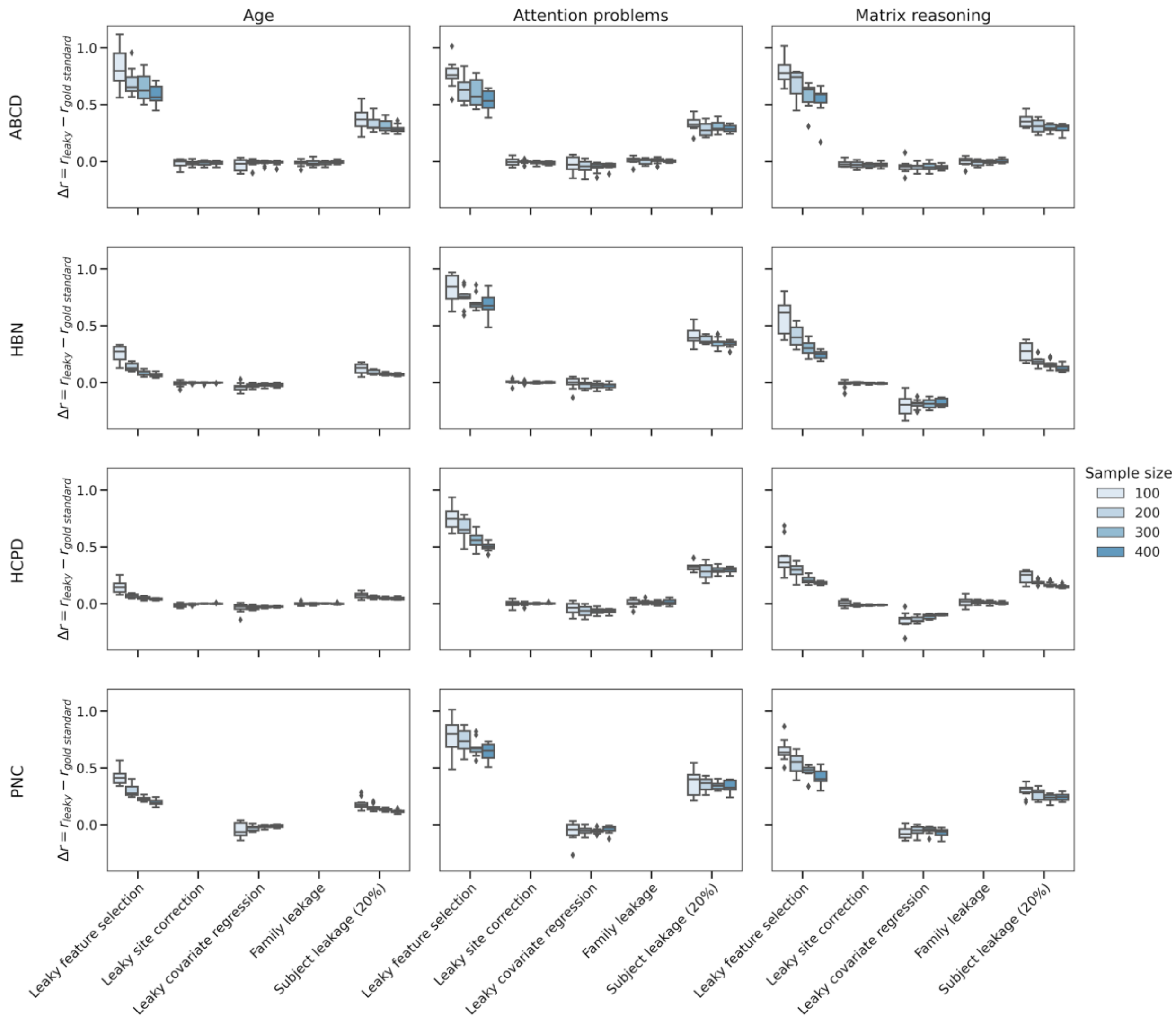

Supplementary Figure 9. Difference in median performances across 10 random seeds of 5-fold cross-validation, related to Figure 9. Rows represent the dataset, and columns show the phenotype. For each leakage type (x-axis), there are four results (N=100, 200, 300, 400). For each sample size, we repeated 10 random seeds of resampling for 10 iterations of 5-fold cross-validation. Boxplot elements were defined as follows: the center line is the median across 10 iterations; box limits are the upper and lower quartiles; whiskers are 1.5x the interquartile range; points are outliers. ABCD: Adolescent Brain Cognitive Development; HBN: Healthy Brain Network; HCPD: Human Connectome Project Development; PNC: Philadelphia Neurodevelopmental Cohort.

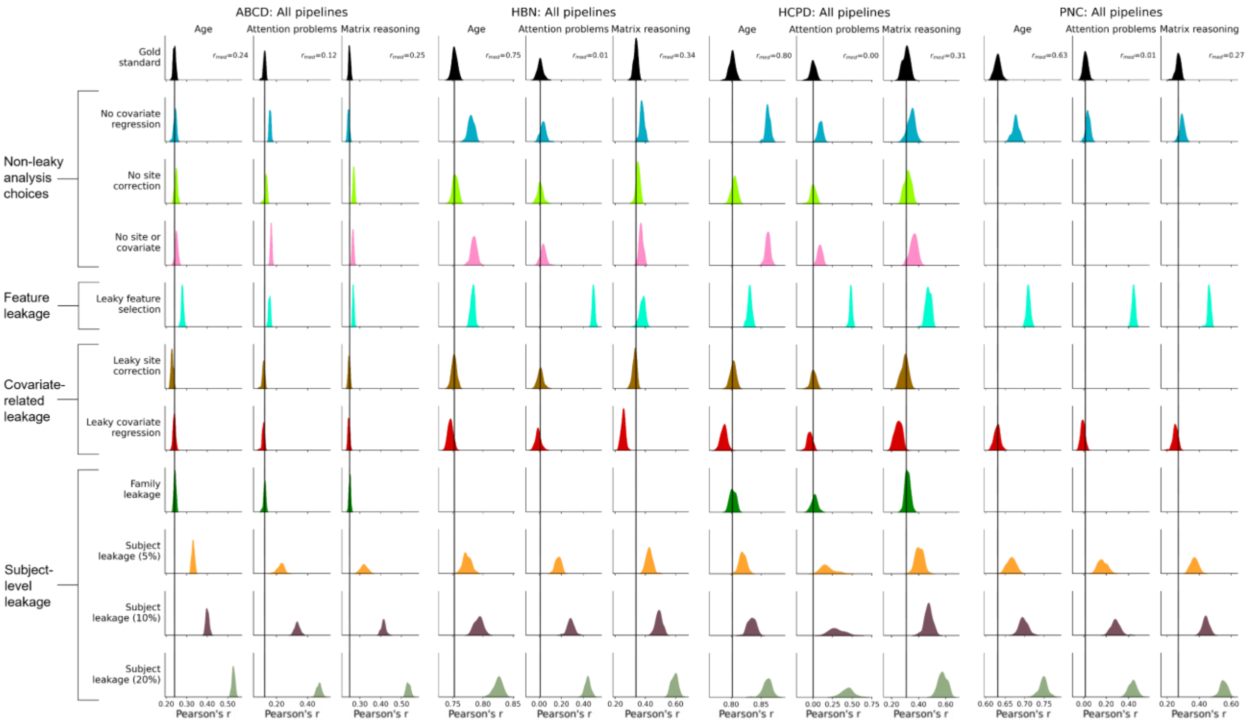

Supplementary Figure 10. Evaluation of leakage types with *r* and SVR. The rows show different leakage types, and, within each dataset, the columns show different phenotypes. The black bar represents the median performance of the "gold standard" models across random iterations, and the histograms show prediction performance across 100 iterations of 5-fold cross-validation. ABCD: Adolescent Brain Cognitive Development; HBN: Healthy Brain Network; HCPD: Human Connectome Project Development; PNC: Philadelphia Neurodevelopmental Cohort.

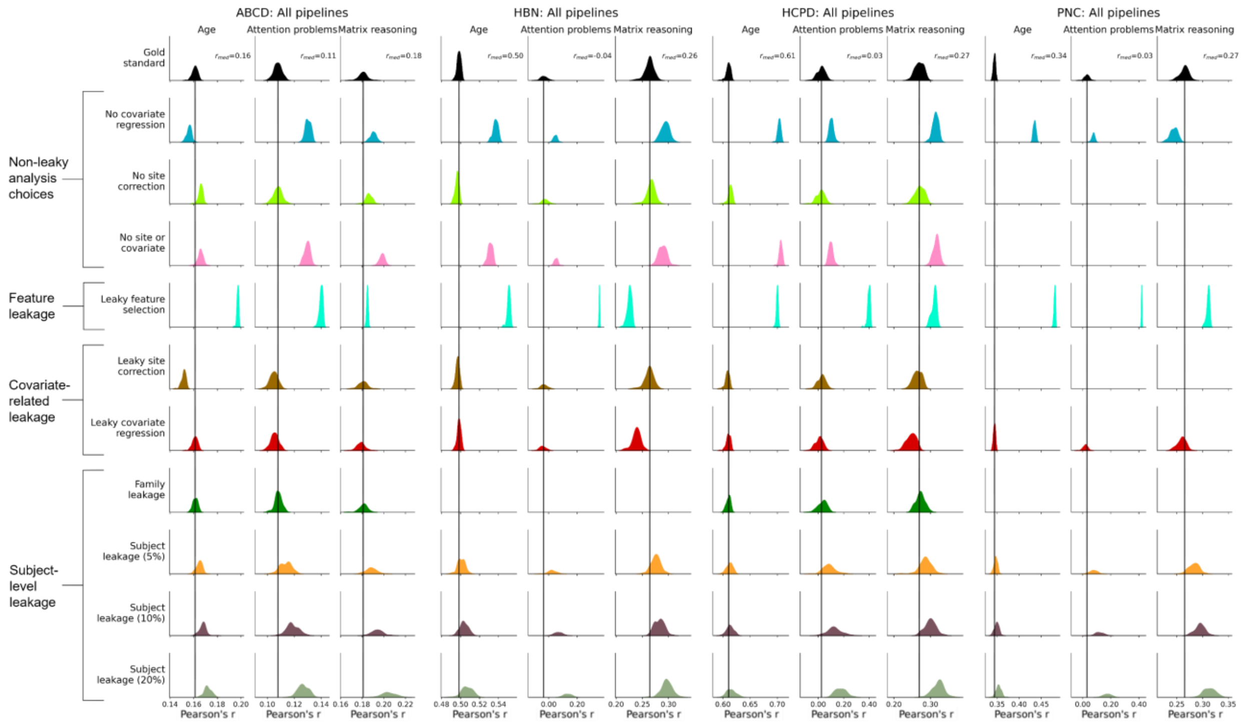

Supplementary Figure 11. Evaluation of leakage types with *r* and CPM. The rows show different leakage types, and, within each dataset, the columns show different phenotypes. The black bar represents the median performance of the "gold standard" models across random iterations, and the histograms show prediction performance across 100 iterations of 5-fold cross-validation. ABCD: Adolescent Brain Cognitive Development; HBN: Healthy Brain Network; HCPD: Human Connectome Project Development; PNC: Philadelphia Neurodevelopmental Cohort.

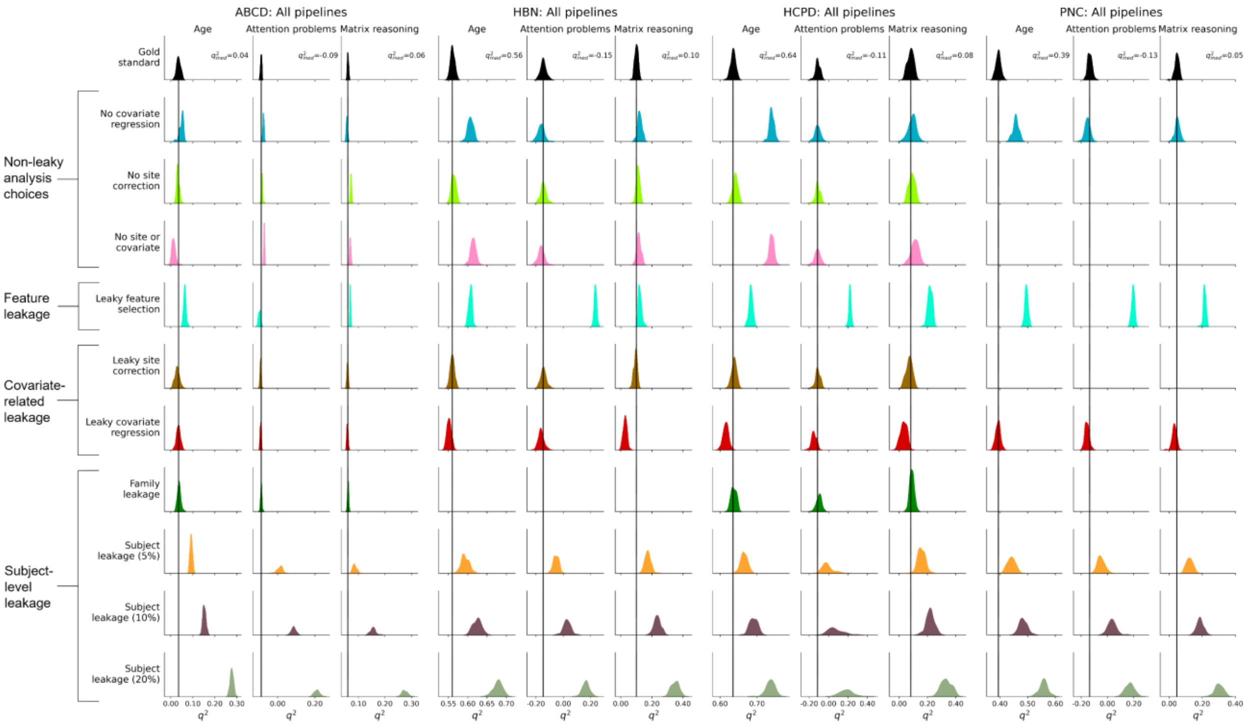

Supplementary Figure 12. Evaluation of leakage types with q^2 and SVR. The rows show different leakage types, and, within each dataset, the columns show different phenotypes. The black bar represents the median performance of the “gold standard” models across random iterations, and the histograms show prediction performance across 100 iterations of 5-fold cross-validation. ABCD: Adolescent Brain Cognitive Development; HBN: Healthy Brain Network; HCPD: Human Connectome Project Development; PNC: Philadelphia Neurodevelopmental Cohort.

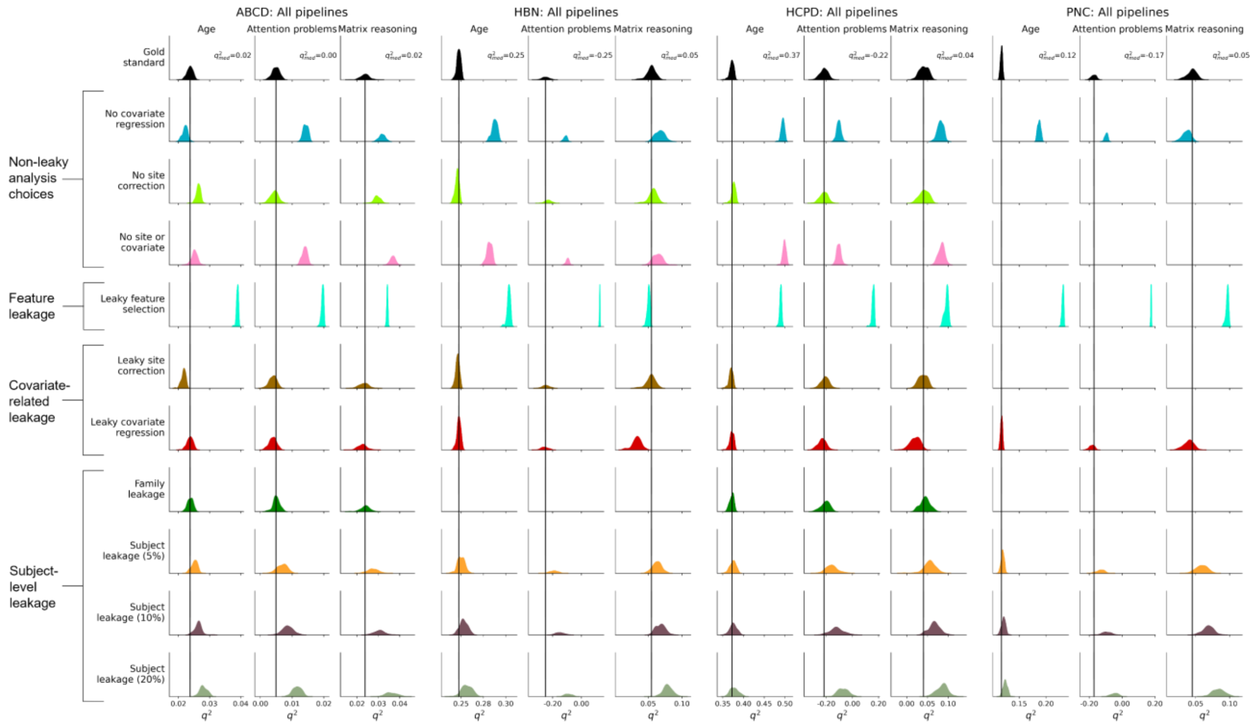

Supplementary Figure 13. Evaluation of leakage types with q^2 and CPM. The rows show different leakage types, and, within each dataset, the columns show different phenotypes. The black bar represents the median performance of the “gold standard” models across random iterations, and the histograms show prediction performance across 100 iterations of 5-fold cross-validation. ABCD: Adolescent Brain Cognitive Development; HBN: Healthy Brain Network; HCPD: Human Connectome Project Development; PNC: Philadelphia Neurodevelopmental Cohort.

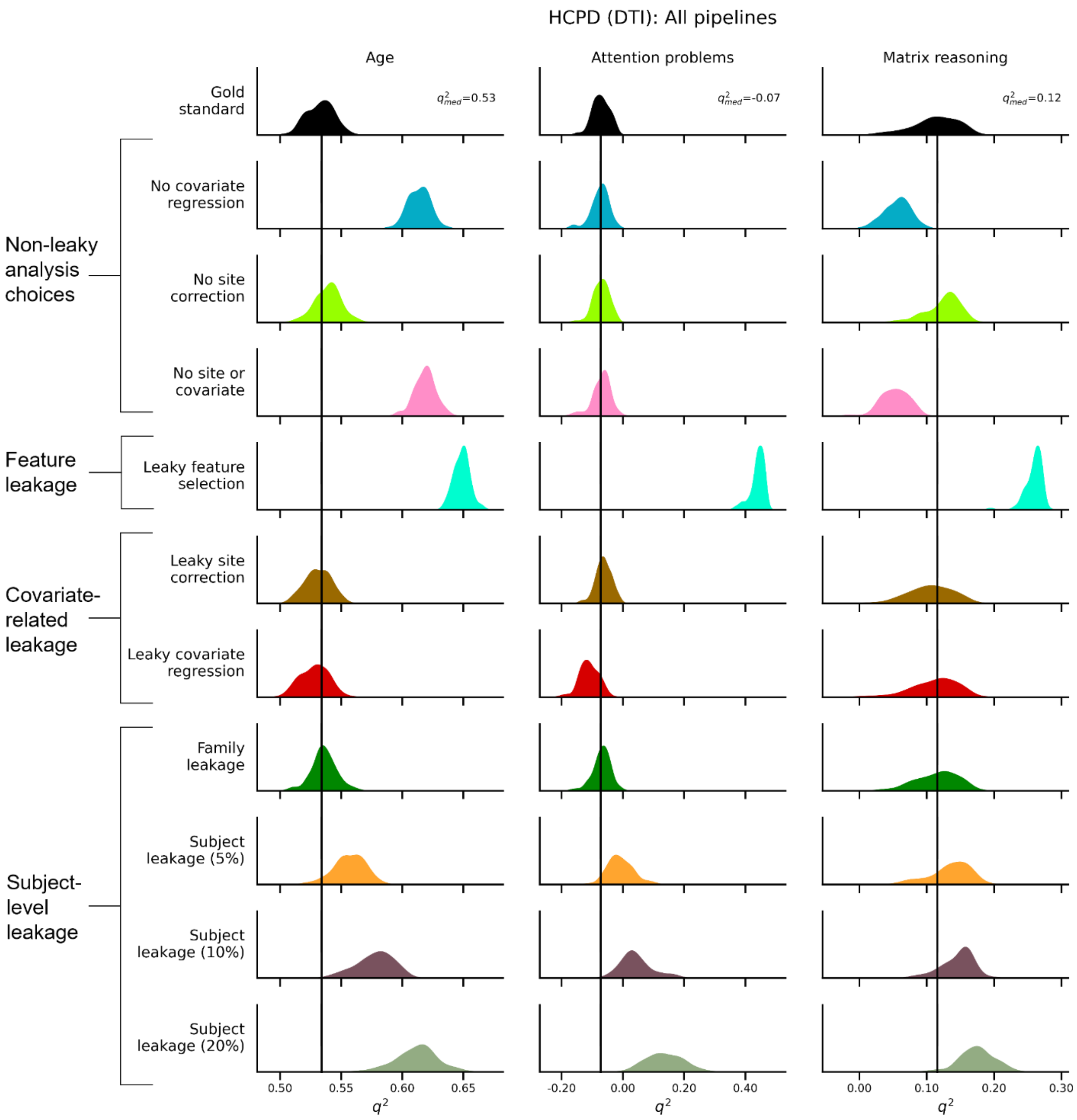

Supplementary Figure 14. Evaluation of leakage types for matrix reasoning, attention problems, and age prediction in structural connectomes using q^2, related to Figure 10. The rows show different leakage types, and the columns show different phenotypes. The black bar represents the median performance of the "gold standard" models across random iterations, and the histograms show prediction performance across 100 iterations of 5-fold cross-validation. HCPD: Human Connectome Project Development; DTI: diffusion tensor imaging.
