# Supplementary material for: Data leakage inflates prediction performance in connectome-based machine learning models
Source: Nat Commun. 2024 Feb 28;15:1829. doi: 10.1038/s41467-024-46150-w (PMC10901797; doi:10.1038/s41467-024-46150-w)
Supplement: Supplementary file 3 — Reporting Summary [file 41467_2024_46150_MOESM3_ESM.pdf]

nature portfolio

Corresponding author(s): Matthew Rosenblatt

Last updated by author(s): Jan 26, 2024

Reporting Summary

Nature Portfolio wishes to improve the reproducibility of the work that we publish. This form provides structure for consistency and transparency in reporting. For further information on Nature Portfolio policies, see our Editorial Policies and the Editorial Policy Checklist.

Statistics

For all statistical analyses, confirm that the following items are present in the figure legend, table legend, main text, or Methods section.

n/a	Confirmed	
[ ]	[x]	The exact sample size (*n*) for each experimental group/condition, given as a discrete number and unit of measurement
[ ]	[x]	A statement on whether measurements were taken from distinct samples or whether the same sample was measured repeatedly
[x]	[ ]	The statistical test(s) used AND whether they are one- or two-sided<br>*Only common tests should be described solely by name; describe more complex techniques in the Methods section.*
[ ]	[x]	A description of all covariates tested
[x]	[ ]	A description of any assumptions or corrections, such as tests of normality and adjustment for multiple comparisons
[ ]	[x]	A full description of the statistical parameters including central tendency (e.g. means) or other basic estimates (e.g. regression coefficient) AND variation (e.g. standard deviation) or associated estimates of uncertainty (e.g. confidence intervals)
[x]	[ ]	For null hypothesis testing, the test statistic (e.g. *F*, *t*, *r*) with confidence intervals, effect sizes, degrees of freedom and *P* value noted<br>*Give P values as exact values whenever suitable.*
[x]	[ ]	For Bayesian analysis, information on the choice of priors and Markov chain Monte Carlo settings
[x]	[ ]	For hierarchical and complex designs, identification of the appropriate level for tests and full reporting of outcomes
[ ]	[x]	Estimates of effect sizes (e.g. Cohen's *d*, Pearson's *r*), indicating how they were calculated

Our web collection on statistics for biologists contains articles on many of the points above.

Software and code

Policy information about availability of computer code

Data collection	No data collection was performed in this study. Preprocessing was carried out using BioImage Suite, which is freely available here: (https://medicine.yale.edu/bioimaging/suite/).
Data analysis	Python 3.7 was used to analyze the data. Additional packages included numpy 1.24.3, pandas 2.0.3, scikit-learn 1.2.2, and scipy 1.10.1. All analysis code is available on GitHub: https://github.com/mattrosenblatt7/leakage_neuroimaging. This includes a link to a Google Colaboratory session, where an environment is set up to re-create all plots (based on a processed .csv file of the results from our scripts).

For manuscripts utilizing custom algorithms or software that are central to the research but not yet described in published literature, software must be made available to editors and reviewers. We strongly encourage code deposition in a community repository (e.g. GitHub). See the Nature Portfolio guidelines for submitting code & software for further information.

Data

Policy information about availability of data

All manuscripts must include a data availability statement. This statement should provide the following information, where applicable:

- Accession codes, unique identifiers, or web links for publicly available datasets
- A description of any restrictions on data availability
- For clinical datasets or third party data, please ensure that the statement adheres to our policy

Data are available through the Adolescent Brain Cognitive Development Study (22, https://abcdstudy.org/), the Healthy Brain Network Dataset (23, http://fcon_1000.projects.nitrc.org/indi/cmi_healthy_brain_network/), the Human Connectome Project Development Dataset (24, https://www.humanconnectome.org/

study/hcp-lifespan-development/overview), and the Philadelphia Neurodevelopmental Cohort Dataset (25, 26, https://www.med.upenn.edu/bbl/philadelphianeurodevelopmentalcohort.html, accession code: phs000607.v3.p2). The ABCD dataset was downloaded from the NIMH Data Archive (NDA). The HBN dataset was downloaded via the HBN portal on through the Longitudinal Online Research and Imaging System (LORIS). The HCP-Development 2.0 Release dataset was downloaded from the NDA and came from DOI: 10.15154/1520708. The PNC dataset was downloaded via dbGaP (accession code: phs000607.v3.p2). Funding details of these datasets are included in the Acknowledgments section of the manuscript.

Source data (i.e., prediction performance values) that can be used for creating the plots are provided with this work and also available at https://github.com/mattrosenblatt7/leakage_neuroimaging.

Research involving human participants, their data, or biological material

Policy information about studies with human participants or human data. See also policy information about sex, gender (identity/presentation), and sexual orientation and race, ethnicity and racism.

Reporting on sex and gender	We described data on self-reported sex for each dataset in the manuscript, and self-reported sex was included as a covariate.
Reporting on race, ethnicity, or other socially relevant groupings	No race or ethnicity data were reported in this study. However, the original datasets do describe race and ethnicity data.
Population characteristics	ABCD consists of 9-10 year olds in the United States. HBN consists of 5-22 year olds from the greater New York area. HCPD includes healthy participants ages 8-22, with imaging data acquired at four sites across the United States (Harvard, UCLA, University of Minnesota, Washington University in St. Louis). The PNC dataset consists of 8-21 year-olds in the Philadelphia area who received care at the Children's Hospital of Philadelphia. Detailed population descriptions are in the relevant papers for the Adolescent Brain Cognitive Development Study (Casey et al., 2018), the Healthy Brain Network Dataset (Alexander et al., 2017), the Human Connectome Project Development Dataset (Somerville et al., 2018), and the Philadelphia Neurodevelopmental Cohort Dataset (Satterthwaite et al, 2014; Satterthwaite et al., 2016).
Recruitment	Full recruitment procedures are available from the Adolescent Brain Cognitive Development Study (Casey et al., 2018), the Healthy Brain Network Dataset (Alexander et al., 2017), the Human Connectome Project Development Dataset (Somerville et al., 2018), and the Philadelphia Neurodevelopmental Cohort Dataset (Satterthwaite et al, 2014; Satterthwaite et al., 2016).
Ethics oversight	The four datasets used in this study were each supervised by their relevant ethical review boards. Informed consent was obtained by the relevant data collection teams in the four public datasets. We have a Yale IRB exception (HIC: 2000023326) to use open-source neuroimaging data.

Note that full information on the approval of the study protocol must also be provided in the manuscript.

Field-specific reporting

Please select the one below that is the best fit for your research. If you are not sure, read the appropriate sections before making your selection.

☒ Life sciences ☐ Behavioural & social sciences ☐ Ecological, evolutionary & environmental sciences

For a reference copy of the document with all sections, see nature.com/documents/nr-reporting-summary-flat.pdf

Life sciences study design

All studies must disclose on these points even when the disclosure is negative.

Sample size	We did not pre-determine sample size since we did not collect data. Instead, four large, open-source neuroimaging datasets were selected for this study. These datasets were selected because they are relatively large and widely used developmental neuroimaging datasets. Four datasets were chosen because replication across multiple independent datasets improves reproducibility and allows for studying similarities and differences in the effects of leakage across a wider range of participants.
Data exclusions	Data were excluded for missing behavioral measurements, high motion (framewise displacement >0.2 mm), or missing coverage in the fMRI scan.
Replication	We demonstrated our findings across four datasets and three types of machine learning models. In addition, we performed sensitivity analysis based on sample size. In general, the trends in the effects of leakage were similar across all four datasets, though there were some minor differences. For example, while excluding site correction had little to no effect in HCPD or HBN, there was a small effect in ABCD. In addition, the dataset with the largest sample size (ABCD), was least affected by leaky feature selection
Randomization	For prediction, data were randomly split with 5-fold cross-validation (which was repeated for 100 random splits). For sample size analysis, data were randomly resampled without replacement.
Blinding	No group allocation (continuous or ordinal measures were used)

Reporting for specific materials, systems and methods

We require information from authors about some types of materials, experimental systems and methods used in many studies. Here, indicate whether each material, system or method listed is relevant to your study. If you are not sure if a list item applies to your research, read the appropriate section before selecting a response.

Materials & experimental systems

n/a	Involved in the study
[x]	[ ] Antibodies
[x]	[ ] Eukaryotic cell lines
[x]	[ ] Palaeontology and archaeology
[x]	[ ] Animals and other organisms
[x]	[ ] Clinical data
[x]	[ ] Dual use research of concern
[x]	[ ] Plants

Methods

n/a	Involved in the study
[x]	[ ] ChIP-seq
[x]	[ ] Flow cytometry
[ ]	[x] MRI-based neuroimaging

Plants

Seed stocks: N/A ("Plants" is checked as n/a above)

Novel plant genotypes: N/A ("Plants" is checked as n/a above)

Authentication: N/A ("Plants" is checked as n/a above)

Magnetic resonance imaging

Experimental design

Design type: Resting-state fMRI

Design specifications: Details are published in the Adolescent Brain Cognitive Development Study (Casey et al., 2018), the Healthy Brain Network Dataset (Alexander et al., 2017), the Human Connectome Project Development Dataset (Somerville et al., 2018), and the Philadelphia Neurodevelopmental Cohort Dataset (Satterthwaite et al, 2014; Satterthwaite et al., 2016).

Behavioral performance measures: Age: age in years

Attention problems: Child Behavior Checklist (CBCL) (29) Attention Problems Raw Score (Achenbach and Ruffle, 2000) was used for ABCD, HBN, and HCPD. Structured Interview for Prodromal Symptoms (Miller et al., 2003) Trouble with Focus and Attention Severity Scale (SIP001, accession code: phv00194672.v2.p2) was used for PNC.

Matrix reasoning: Wechsler Intelligence Scale for Children (WISC-V) (Wechsler, 2014) Matrix Reasoning Total Raw Score was used for ABCD, HBN, and HCPD. Penn Matrix Reasoning (Bilker et al., 2012; Moore et al., 2015) Total Raw Score (PMAT_CR, accession code: phv00194834.v2.p2) was used for PNC.

Acquisition

Imaging type(s): functional MRI

Field strength: All datasets were collected at 3T, except a portion of the Healthy Brain Network dataset was collected at 1.5T

Sequence & imaging parameters: Details of the sequence and imaging parameters for each of the four datasets used in this study are published in the relevant papers: the Adolescent Brain Cognitive Development Study (Casey et al., 2018), the Healthy Brain Network Dataset (Alexander et al., 2017), the Human Connectome Project Development Dataset (Somerville et al., 2018), and the Philadelphia Neurodevelopmental Cohort Dataset (Satterthwaite et al, 2014; Satterthwaite et al., 2016).

Area of acquisition: Whole brain scan

Diffusion MRI: [x] Used [ ] Not used

Parameters: As described in the HCPD paper (Somerville et al., 2014): "185 directions on 2 shells of b=1500 and 3000 s/mm2, along with b=0 s/mm2 images"

Preprocessing

Preprocessing software: Preprocessing was carried out using BioImage Suite, which is freely available here: (https://medicine.yale.edu/bioimaging/suite/).

Normalization: nonlinear normalization into MNI space

Normalization template: MNI304

Noise and artifact removal: Several covariates of no interest were regressed from participants' functional data including linear and quadratic drifts, mean cerebrospinal fluid signal, mean white matter signal, and mean global signal. For additional control of possible motion-related confounds, a 24-parameter motion model (including six rigid body motion parameters, six temporal derivatives, and these terms squared) was regressed from the data. The data were temporally smoothed with a Gaussian filter (approximate cutoff frequency=0.12 Hz).

Volume censoring: Subjects with >0.2 mm mean framewise displacement were excluded

Statistical modeling & inference

Model type and settings: Predictive modeling with three different methods: 1) ridge regression (https://scikit-learn.org/stable/modules/generated/sklearn.linear_model.Ridge.html), 2) support vector regression (https://scikit-learn.org/stable/modules/generated/sklearn.svm.SVR.html), and 3) connectome-based predictive modeling (Shen et al., 2017).

For an additional family analysis, we used a random forest (https://scikit-learn.org/stable/modules/generated/sklearn.ensemble.RandomForestRegressor.html) with 10 estimators. A grid search was performed varying the maximum depth (3, 5, 7, 9).

Effect(s) tested: We tested the prediction of age, attention, and matrix reasoning from functional connectivity data.

Specify type of analysis: [x] Whole brain [ ] ROI-based [ ] Both

Statistic type for inference (See Eklund et al. 2016): We reported prediction performance via Pearson r and cross-validation R^2 ("q^2") (see Scheinost et al., 2019; Poldrack, Huckins, and Varoquaux, 2020).

Correction: Since comparing many models (>500 pipelines total, each repeated for 100 iterations) to each other would require many permutation tests, comparisons with P values was not practical. Instead, we show the distribution of prediction performance for each leakage type across repeats of k-fold cross-validation.

Models & analysis

n/a	Involved in the study
[ ]	[x] Functional and/or effective connectivity
[x]	[ ] Graph analysis
[ ]	[x] Multivariate modeling or predictive analysis

Functional and/or effective connectivity: Pearson correlation followed by the Fisher transform

Multivariate modeling and predictive analysis: We used 5-fold cross-validation to evaluate the three models described above: ridge regression, support vector regression, and connectome-based predictive modeling. Where applicable, data were split into cross-validation groups based on their family structure. Within the cross-validation folds (unless specifically testing for a case of leakage), covariates (age, head motion, sex, acquisition site) were regressed from the functional connectivity data, and then the top 5% of features most significantly correlated with the phenotypic variable were selected. After this, the model was fit to predict the behavioral/phenotypic measure from the functional connectivity features. Furthermore, we performed a grid search over the L2 regularization parameter (10^{-3,-2,-1,0,1,2,3}), with the chosen model being the one with the highest Pearson's correlation value r in the nested folds.
